# Sub-Tenon Block with Bolus-Free Dexmedetomidine Sedation for Penetrating Keratoplasty: A Retrospective Clinical Case Series of 50 High-Risk Patients

**DOI:** 10.3390/life16061019

**Published:** 2026-06-17

**Authors:** Margita Lucic, Borivoje Savic, Jelena Kostic, Sanja Petrovic Pajic, Tiana Petrovic, Dolika D. Vasovic, Tanja Kalezic

**Affiliations:** 1 Center for Anesthesiology and Resuscitation, University Clinical Center of Serbia, 11000 Belgrade, Serbia; margita.84@gmail.com; 2 University Clinical Centre of Serbia, University Eye Hospital, Pasterova 2, 11000 Belgrade, Serbia; 3 Faculty of Medicine, University of Belgrade, 11000 Belgrade, Serbia

**Keywords:** penetrating keratoplasty, corneal transplantation, sub-Tenon block, dexmedetomidine, regional anesthesia, elderly, hemodynamic stability, procedural sedation

## Abstract

**Background**: Penetrating keratoplasty (PK) is a technically demanding corneal transplant procedure frequently performed in elderly patients with substantial systemic comorbidities. In this population, an anesthetic strategy that ensures hemodynamic stability, cooperative sedation, adequate analgesia, and preserved spontaneous ventilation is highly desirable. Dexmedetomidine, a highly selective alpha2-adrenergic agonist, provides “cooperative” sedation with minimal risk of respiratory depression and additional sympatholytic benefits. **Methods**: This single-center retrospective observational case series included 50 consecutive patients (American Society of Anesthesiologists [ASA] II–III, age 50–90 years) undergoing PK under sub-Tenon block combined with continuous dexmedetomidine infusion. Dexmedetomidine was administered without a loading bolus at 0.7 mcg/kg/h for 10–15 min, then reduced to 0.5 mcg/kg/h, targeting a Ramsay Sedation Scale (RSS) score of 2–3. The sub-Tenon block was performed using a mixture of levobupivacaine 0.5% and lidocaine 2% (3–5 mL). Heart rate (HR), mean arterial pressure (MAP), oxygen saturation (SpO_2_) and RSS were recorded in nine predefined perioperative phases. Data were analyzed descriptively. **Results**: The mean age was 72 ± 9 years; 52% of patients were ASA III. Hypertension was present in all patients; 30% had cardiovascular disease, 28% diabetes mellitus type II, and 30% chronic obstructive pulmonary disease. Progressive, controlled bradycardia was observed (mean HR decreased from 76 to 57 beats/min during graft transplantation), while MAP gradually decreased from hypertensive baseline values (150–160 mmHg) to an optimal intraoperative range of 115–130 mmHg, without episodes of clinically significant hypotension. SpO_2_ remained stable at 98–99% throughout all phases, with no episodes of desaturation or need for airway intervention or supplemental oxygen. Target sedation (RSS 2–3) was achieved in all patients (median RSS 3), with preserved spontaneous breathing and cooperation. Sub-Tenon block-related bulging occurred in 6% of cases. No episodes of clinically significant bradycardia, malignant arrhythmia, respiratory compromise, or need to discontinue dexmedetomidine were recorded. No opioids or non-steroidal analgesics were required intraoperatively or in the early postoperative period. **Conclusions**: The combination of sub-Tenon block and continuous dexmedetomidine sedation without a loading bolus represents a hemodynamically stable and respiratory-safe anesthetic strategy for PK in elderly, high-risk patients. These preliminary, hypothesis-generating findings suggest that the protocol provides stable surgical conditions and a favorable safety profile, justifying future prospective randomized controlled trials to establish its comparative efficacy against general anesthesia or standard sedative regimens.

## 1. Introduction

Corneal transplantation remains one of the most successful tissue transplantation procedures and is the primary therapeutic option for advanced corneal opacities, scars, and ectatic disorders [1,2,3,4]. Among its surgical variants, penetrating keratoplasty (PK) involves replacement of the central or paracentral diseased corneal segment while preserving the peripheral host rim near the limbus. This technique provides improved structural stability, may reduce postoperative astigmatism, and facilitates more predictable graft integration compared with full-thickness procedures [5,6,7,8].

From a surgical standpoint, PK requires absolute ocular immobility, stable intraocular pressure (IOP), and consistent anterior chamber depth throughout the procedure. Even minor fluctuations in IOP or involuntary eye movements may compromise the safety of intraocular manipulation, suture placement, or graft centration, potentially affecting long-term graft survival [9,10,11,12,13,14,15].

The population undergoing PK is typically elderly and burdened with systemic comorbidities such as arterial hypertension, ischemic heart disease, diabetes mellitus, and chronic obstructive pulmonary disease (COPD) [2,10,13]. In such patients, general anesthesia may provoke undesirable hemodynamic fluctuations during induction and emergence, alter IOP through positive-pressure ventilation, and increase the risks of postoperative delirium and cognitive dysfunction [16,17,18,19,20,21,22,23,24]. Consequently, anesthetic strategies combining regional ophthalmic anesthesia with procedural sedation have gained increasing attention as safer alternatives.

The sub-Tenon block has emerged as a preferred regional technique in ophthalmic anesthesia, providing reliable analgesia and akinesia with a lower incidence of serious complications—such as globe perforation, retrobulbar hemorrhage, intravascular injection, and optic nerve injury—compared with retrobulbar and peribulbar techniques [17,18,19,20,21,22,23,25,26,27]. Several comparative studies have demonstrated favorable outcomes with the sub-Tenon approach, including excellent patient comfort, high rates of adequate akinesia, and a strong overall safety profile [25]. For corneal transplantation, where ocular stability and tight IOP control are essential, these advantages make the sub-Tenon block particularly suitable.

Dexmedetomidine, a highly selective alpha2-adrenergic agonist, provides sedative, anxiolytic, and modest analgesic effects while preserving spontaneous ventilation. Its predictable sympatholytic profile contributes to reduced IOP, blunted hemodynamic responses, and the unique phenomenon of cooperative sedation, in which patients remain calm yet easily arousable and capable of following verbal instructions [28,29,30,31,32,33,34,35,36,37]. Recent ophthalmic studies have reported favorable surgical conditions and high surgeon satisfaction when dexmedetomidine is used for sedation during regional ophthalmic anesthesia [31,33,34,38,39,40]. However, data on the combined use of the sub-Tenon block and continuous, bolus-free dexmedetomidine sedation specifically for PK in high-risk elderly patients remain limited.

The primary aim of this retrospective clinical case series was to evaluate the hemodynamic and respiratory stability of a standardized anesthetic protocol combining sub-Tenon anesthesia with continuous dexmedetomidine infusion in patients undergoing PK. Secondary aims were to assess: (i) sedation quality using the Ramsay Sedation Scale (RSS), (ii) the need for supplemental analgesia, and (iii) the incidence of block- or sedation-related adverse events. A narrative review of the relevant literature is provided to contextualize the findings and highlight the potential of this anesthetic approach in corneal transplantation.

## 2. Materials and Methods

### 2.1. Study Design and Patients

This is a single-center, retrospective observational case series including 50 consecutive patients who underwent penetrating keratoplasty (PK) under sub-Tenon block with dexmedetomidine (Dexmedetomidine Pharmidea®, PHARMIDEA SIA, Olaine, Latvia) sedation at the University Eye Hospital, University Clinical Center of Serbia, between January 2024 and December 2025.

### 2.2. Inclusion Criteria

Age ≥ 50 years;

American Society of Anesthesiologists (ASA) physical status II–III;

Clear clinical indication for PK;

Sub-Tenon block combined with dexmedetomidine-based procedural sedation as the primary anesthetic technique.

Patients who required conversion to general anesthesia or received alternative sedative/analgesic regimens (e.g., propofol, midazolam, or remifentanil) were excluded from this analysis.

### 2.3. Ethics Statement

The study was conducted in accordance with the Declaration of Helsinki and approved by the Institutional Review Board of University Clinical Center of Serbia, Eye Hospital (protocol code 1880/33, date 25 December 2025). The requirement for individual informed consent was waived due to the retrospective, non-interventional design of the study and the use of completely anonymized clinical data.

### 2.4. Anesthetic Protocol

All patients received standard oral premedication with bromazepam 1.5–3 mg approximately 60 min before transfer to the operating room. Dexmedetomidine sedation was initiated 10 min before performing the sub-Tenon block and was maintained continuously throughout the surgical procedure.

Dexmedetomidine was prepared by diluting 200 mcg of the drug in 50 mL of 0.9% NaCl (final concentration 4 mcg/mL). To prevent abrupt hemodynamic drops, no loading bolus was administered. The initial infusion rate was set at 0.7 mcg/kg/h for the first 10–15 min to facilitate block performance and the start of surgery, and was visited down to a maintenance rate of 0.5 mcg/kg/h. The infusion was titrated to maintain a target sedation depth of RSS 2–3 (calm, cooperative, and responsive to verbal commands). No opioids or non-steroidal anti-inflammatory drugs (NSAIDs) were administered intraoperatively or in the immediate postoperative period in the recovery room.

### 2.5. Sub-Tenon Block Technique

All ophthalmic blocks were performed under strict aseptic conditions by an experienced anesthesiologist or ophthalmologist, with the patient in the supine position, following approximately 10 min of initial dexmedetomidine infusion.

After preparing the periocular skin with povidone–iodine and disinfecting the conjunctival sac, local anesthetic drops (tetracaine 0.5%) were instilled. A small conjunctival incision was made in the inferonasal quadrant approximately 5–7 mm from the limbus using blunt Westcott scissors to expose the sclera. A curved, blunt sub-Tenon cannula (23–25 G) was carefully advanced into the sub-Tenon space following the contour of the globe. After confirming free advancement without resistance or pain, a mixture of levobupivacaine 0.5% and lidocaine 2% (total volume 3–5 mL, adjusted based on individual ocular anatomy) was injected slowly. Gentle digital massage of the eyelids was applied for 2–3 min to enhance even spread towards the posterior perioperative segment.

Passive diffusion in the sub-Tenon space provides access to:

Sensory fibers of the ophthalmic division of the trigeminal nerve (V1), particularly the nasociliary nerve, resulting in effective corneal and anterior segment analgesia.

Motor fibers of the oculomotor, trochlear and abducens nerves, producing complete ocular akinesia by blocking extraocular muscle innervation.

Autonomic fibers, reducing sympathetic tone to the ciliary body and dampening IOP fluctuations and the oculocardiac reflex [17,18,19,20,21,22,23,25,26,27,41,42].

Compared with retrobulbar and peribulbar blocks, the sub-Tenon approach reduces the risk of globe perforation, intravascular injection, optic nerve trauma, and sudden increases in IOP [17,18,19,20,21,22,23,25,26,27].

### 2.6. Perioperative Monitoring and Outcome Measures

Standard non-invasive monitoring was established upon arrival in the operating room and included continuous lead II electrocardiography (ECG), non-invasive blood pressure (NIBP), and pulse oximetry (SpO_2_). Hemodynamic and respiratory parameters, alongside the Ramsay Sedation Scale (RSS) score, were recorded across nine predefined clinical phases:

Baseline—45 min before surgery in the premedication area;
On operating table, before starting sedation;
Start of dexmedetomidine infusion;
Sub-Tenon block performance;
Start of surgery;
Corneal manipulation;
Graft transplantation;
End of surgery;
Early postoperative period—30 min after surgery in the recovery room.

Primary Outcomes:

**Hemodynamic stability:** Trends in HR and MAP across phases. *Clinically significant bradycardia* was defined as a heart rate < 45 beats/min, or any drop in HR accompanied by signs of hypoperfusion or hemodynamic instability requiring pharmacological intervention (e.g., atropine). *Clinically significant hypotension* was defined as a mean arterial pressure (MAP) less than 60 mmHg, or a decrease in systolic blood pressure of greater than 30% from the patient’s baseline value, requiring fluid boluses or vasopressor support (e.g., ephedrine).

Respiratory stability: SpO_2_ trends, absence of desaturation (SpO_2_ < 92%), apnea, hypoventilation, or need for airway intervention or oxygen supplementation.

Secondary Outcomes:

Sedation quality assessed by the Ramsay Sedation Scale (RSS) [43,44,45,46], with an optimal target of 2–3.

Need for additional sedatives or analgesics.

Incidence of sub-Tenon-related complications (e.g., bulging/globe tension).

Patient and surgeon satisfaction (noted qualitatively from chart documentation).

### 2.7. Statistical Analysis

Given the descriptive and exploratory character of this retrospective case series, no formal hypothesis testing was undertaken. Continuous variables are presented as mean ± standard deviation (SD), range, or median, as appropriate. Categorical variables are summarized as absolute numbers and percentages. Data were analyzed using IBM SPSS Statistics for Windows, version 26.0 (IBM Corp., Armonk, NY, USA); no advanced modeling or multivariable analysis was attempted due to sample size and study design.

## 3. Results

### 3.1. Patient Characteristics

A total of 50 patients were included. Demographic characteristics are summarized in Table 1. The mean age was 72 ± 9 years (range 50–90), with a median of 72 years. The sex distribution was balanced (26 males, 24 females). ASA class II and III patients represented 48% and 52% of the cohort, respectively.

Comorbidities are detailed in Table 2. Hypertension was present in all patients (100%). Thirty percent had cardiovascular disease, including prior myocardial infarction in 12% and previous coronary artery bypass grafting in 6%. Diabetes mellitus type II was present in 28% and COPD in 30% of patients. Hypothyroidism occurred in 8%, and 18% of patients were overweight or obese (BMI > 25 kg/m^2^). This profile reflects a typical geriatric, high-risk PK population.

### 3.2. Sub-Tenon Block and Sedation Characteristics

Characteristics of the block and sedation are summarized in Table 3. All patients underwent PK under a sub-Tenon block with levobupivacaine 0.5% + lidocaine 2% (3–5 mL). Ocular akinesia was achieved within 5–10 min in all cases. Sub-Tenon-related bulging (increased globe tension) was observed in 3 patients (6%), none of whom required modification of the surgical plan or additional interventions.

Dexmedetomidine infusion was initiated without a loading bolus at 0.7 mcg/kg/h and reduced to 0.5 mcg/kg/h after 10–15 min. The target sedation level (RSS 2–3) was achieved and maintained in all patients. No patient required adjunctive opioids or non-steroidal analgesics intraoperatively or in the early postoperative period in the recovery room.

### 3.3. Hemodynamic and Respiratory Parameters

Hemodynamic data across the nine perioperative phases are shown in Table 4. A progressive, controlled decrease in heart rate was observed, with mean HR declining from 76 ± 8 beats/min at baseline to 57 ± 6 beats/min during graft transplantation, consistent with the expected sympatholytic effect of dexmedetomidine, but without symptoms or need for pharmacological intervention.

Baseline MAP values, reflecting prevalent hypertension (150–160 mmHg), gradually decreased to an optimal intraoperative range of 115–130 mmHg, with further stabilization toward the end of surgery and a modest increase in the early recovery phase. No episodes of clinically significant hypotension requiring intervention were recorded.

Respiratory parameters remained remarkably stable, with SpO_2_ values of 98–99% throughout all perioperative phases. No episodes of desaturation, apnea, or hypoventilation occurred, and no airway maneuvers or oxygen supplementation were required.

### 3.4. Sedation Quality

The Ramsay Sedation Scale (RSS) was used to titrate and monitor sedation depth. The target level of RSS 2–3 (calm, cooperative patient, responding to verbal commands) was consistently achieved; the median RSS was 3 (Table 5). No episode of excessive sedation (RSS ≥ 4) was documented. All patients remained spontaneously breathing, responsive to verbal stimuli, and comfortable throughout surgery.

### 3.5. Safety Outcomes

The safety profile is summarized in Table 6. No episodes of clinically significant bradycardia requiring treatment, clinically significant hypotension, or malignant arrhythmias were observed. No respiratory complications, desaturation, or need for airway intervention occurred. There was no need to discontinue dexmedetomidine infusion or provide rescue analgesics intraoperatively or in the early postoperative period. The overall tolerability of the regimen was excellent, and early postoperative recovery was rapid, with patients awake, communicative, and hemodynamically stable in the recovery room.

## 4. Discussion

In this retrospective case series of 50 elderly, comorbid patients undergoing penetrating keratoplasty, we found that a standardized protocol combining sub-Tenon block with continuous dexmedetomidine sedation (without loading bolus) provided excellent surgical conditions, predictable hemodynamic control, and remarkable respiratory safety.

### 4.1. Regional Anesthesia and Sub-Tenon Block in Ophthalmic Surgery

Regional techniques have increasingly replaced general anesthesia in many ophthalmic procedures due to lower systemic risk, faster recovery and improved cost-effectiveness [16,17,18,19,20,21,22,23,24,26,27,42]. The sub-Tenon block, popularized as a safer alternative to retrobulbar and peribulbar injections, provides reliable analgesia and akinesia while minimizing the risk of globe perforation, optic nerve injury, and intravascular injection [17,18,19,20,21,22,23,25,26,27]. Our low incidence of block-related bulging (6%) is in line with published rates, typically ranging between 5–12%, and did not influence surgical management.

For corneal transplantation, where IOP stability and ocular immobility are crucial, the sub-Tenon block is particularly attractive: it reduces extraocular muscle tone, dampens afferent input from the trigeminal nerve and blunts the oculocardiac reflex [16,17,21,22,23,25,41]. Our findings support these advantages, as we observed no clinically relevant arrhythmias or serious reflex responses.

### 4.2. Dexmedetomidine in Ophthalmic Anesthesia

Dexmedetomidine’s pharmacodynamic profile—sedation, anxiolysis, mild analgesia, sympatholysis, and minimal respiratory depression—makes it ideally suited for procedural sedation in high-risk populations [28,29,30,32,33,34,35,36,37,47,48,49,50]. Several studies have demonstrated its beneficial effects in ophthalmic surgery, including IOP reduction, improved hemodynamic stability, and high patient and surgeon satisfaction [31,33,34,38,39,40].

In our series, the progressive, controlled decrease in HR and MAP from hypertensive baseline values, without any need for rescue therapy, reflects the desired sympatholytic effect in a population where 100% had hypertension and 30% had cardiovascular disease. Rather than inducing hypotension, dexmedetomidine appeared to normalize elevated preoperative pressures. Importantly, we observed no episodes of clinically significant bradycardia or hypotension, which is likely related to the omission of a loading bolus and careful titration of infusion rates.

The absence of respiratory complications in our cohort, including 30% of patients with COPD and a significant number with obesity and cardiovascular disease, confirms the respiratory safety of dexmedetomidine [28,29,30,35,36,47,50]. SpO_2_ remained stable (98–99%) in all phases without supplemental oxygen or airway interventions—an important advantage over benzodiazepines and opioids, which carry a higher risk of respiratory depression, particularly in the elderly [44,49,50]. In the era of opioid-sparing and enhanced recovery strategies, our results provide further evidence that a completely opioid-free, dexmedetomidine-based sedation protocol is a safe, stable, and clinically robust option for complex ophthalmic surgery in high-risk patients.

### 4.3. Sedation Depth and Cooperative Patient Behavior

The target RSS 2–3 is generally considered optimal for ophthalmic surgery performed under regional anesthesia, as patients are calm, not anxious or agitated, yet remain responsive to verbal commands and able to cooperate with positioning and simple instructions [43,44,45,46]. Dexmedetomidine is well known to facilitate such cooperative sedation [33,35,38,39]. In all patients, the median RSS was 3, with no episodes of oversedation. This allowed safe preservation of spontaneous breathing and facilitated communication throughout the procedure.

The lack of need for rescue opioids or additional sedatives underscores the intrinsic analgesic and anxiolytic properties of dexmedetomidine and the effectiveness of the sub-Tenon block. Avoiding opioids is particularly beneficial in elderly populations at risk for postoperative nausea and vomiting (PONV), delirium, and respiratory depression [44,49,50].

### 4.4. Clinical Implications

Our results suggest that, in high-risk elderly patients undergoing PK, the combination of sub-Tenon block and dexmedetomidine sedation:

Provides excellent surgical conditions, with stable ocular tone and akinesia;
Ensures hemodynamic stability without abrupt peaks or drops in blood pressure and HR;
Maintains respiratory safety with preserved spontaneous ventilation;
Minimizes the need for opioids and additional analgesics;
Enables rapid, clear-headed postoperative recovery, reducing the risk of postoperative cognitive disturbances.

In settings where general anesthesia carries substantial risk, this protocol represents a rational and practical strategy that can be integrated into routine practice with modest resource requirements.

### 4.5. Limitations

This study has several important limitations:

Lack of a control group: This was a single-center, retrospective observational study with no concurrent comparison group (e.g., propofol, midazolam, or general anesthesia), which limits causal inference and our ability to claim superiority.

Descriptive analysis only: Due to the exploratory nature of the case series and the sample size, no hypothesis-driven statistical testing or multivariable modeling was attempted.

Lack of quantitative satisfaction and IOP tools: Patient and surgeon satisfaction scores were not evaluated using formalized, validated scales due to the retrospective nature of chart reviews. Similarly, IOP was not quantitatively measured via tonometer; conclusions regarding intraocular pressure stability are based purely on clinical ophthalmic assessment and the absence of intraoperative vitreous bulging or prolapse.

Sample size: The sample size (N = 50), while reasonable for a specialized case series, is insufficient to detect rare adverse events or complications.

Despite these limitations, the consistency of the findings and the high-risk profile of the population strengthen the clinical relevance of our observations and provide a valuable basis for the design of future prospective, randomized studies comparing dexmedetomidine-based sedation with other regimens or general anesthesia in corneal transplantation.

## 5. Conclusions

In this retrospective observational case series of 50 elderly, comorbid patients undergoing penetrating keratoplasty, a standardized combination of sub-Tenon block and continuous bolus-free dexmedetomidine sedation, titrated to RSS 2–3, provided stable surgical conditions, predictable hemodynamic control, and exceptional respiratory safety. The synergistic interaction between the sub-Tenon block and dexmedetomidine sedation appears particularly advantageous in high-risk geriatric patients in whom avoidance of general anesthesia and airway manipulation is desirable.

Because of the retrospective and descriptive nature of this study, these findings should be considered preliminary and hypothesis-generating. They support the clinical viability of this approach in appropriately selected patients and highlight the need for larger, prospective comparative trials to fully define its role and safety profile relative to standard alternative sedative regimens and general anesthesia in modern corneal transplantation.

## Figures and Tables

**Table 1:** Demographic characteristics of the patients.

Parameter	Value
Number of patients	50
Age, mean ± SD (years)	72 ± 9
Age range (years)	50–90
Median age (years)	72
Sex (male/female)	26/24
ASA II/ASA III	24 (48%)/26 (52%)

**Table 2:** Comorbidities.

Comorbidity	Number of Patients *n* (%)
Hypertension	50 (100%)
Cardiovascular disease (any)	15 (30%)
Prior myocardial infarction	6 (12%)
Prior coronary artery bypass graft (CABG)	3 (6%)
Diabetes mellitus type II	14 (28%)
Chronic obstructive pulmonary disease (COPD)	15 (30%)
Hypothyroidism	4 (8%)
Overweight/obesity (BMI ≥ 25 kg/m^2^)	9 (18%)

**Table 3:** Characteristics of the sub-Tenon block and dexmedetomidine sedation.

Parameter	Value
Type of surgery	Penetrating keratoplasty (PK)
Type of anesthesia	Sub-Tenon block + dexmedetomidine sedation
Local anesthetics	Levobupivacaine 0.5% + lidocaine 2%
Total volume of local anesthetics	3–5 mL
Time to akinesia	5–10 min
Bulging after block	3/50 patients (6%)
Premedication	Bromazepam 1.5–3 mg orally
Dexmedetomidine—initial infusion rate	0.7 µg/kg/h (10–15 min)
Dexmedetomidine—maintenance rate	0.5 µg/kg/h
Loading (bolus) dose	Not administered
Target sedation level	RSS 2–3

**Table 4:** Hemodynamic and respiratory parameters during perioperative phases (mean values ± SD).

Phase	Heart Rate (Beats/min)	Blood Pressure * (mmHg)	SpO_2_ (%)
1. Baseline—premedication room	76 ± 8	150 ± 12/75 ± 6	98 ± 1.2
2. On operating table (before sedation)	82 ± 9	155 ± 14/75 ± 7	99 ± 0.8
3. Start of dexmedetomidine infusion	74 ± 7	150 ± 11/70 ± 6	99 ± 0.5
4. Sub-Tenon block performance	77 ± 8	160 ± 15/80 ± 8	98 ± 1.1
5. Start of surgery	72 ± 6	145 ± 12/65 ± 6	99 ± 0.6
6. Corneal manipulation	62 ± 5	130 ± 10/70 ± 5	99 ± 0.4
7. Graft transplantation	57 ± 6	115 ± 9/65 ± 5	99 ± 0.5
8. End of surgery	60 ± 5	110 ± 8/60 ± 5	99 ± 0.7
9. 30 min postoperatively—recovery room	70 ± 7	135 ± 10/75 ± 6	98 ± 0.9

* Blood pressure values are expressed as systolic/diastolic blood pressure (mmHg) and presented as mean ± standard deviation (SD).

**Table 5:** Sedation according to the Ramsay Sedation Scale (RSS).

Parameter	Value
Target sedation level	RSS 2–3
Median sedation level	RSS 3
Episodes of excessive sedation (RSS ≥ 4)	0
Preserved spontaneous breathing	50 patients (100%)
Respiratory complications	0

**Table 6:** Safety profile and complications.

Parameter	Value
Clinically significant bradycardia	Not observed
Clinically significant hypotension	Not observed
Malignant arrhythmias	Not observed
Respiratory complications	None observed
Need to interrupt dexmedetomidine infusion	None observed
Need for supplemental oxygen or airway support	None observed
Need for rescue opioid/NSAID analgesia	None observed

## Data Availability

The raw data supporting the conclusions of this article will be made available by the authors on request.
